# Characterization of the Sucrose Phosphate Phosphatase (SPP) Isoforms from *Arabidopsis thaliana* and Role of the S6PPc Domain in Dimerization

**DOI:** 10.1371/journal.pone.0166308

**Published:** 2016-11-17

**Authors:** Tomás Albi, M. Teresa Ruiz, Pedro de los Reyes, Federico Valverde, José M. Romero

**Affiliations:** Instituto de Bioquímica Vegetal y Fotosíntesis, Universidad de Sevilla and Consejo Superior de Investigaciones Científicas, Seville, Spain; Universidad Miguel Hernández de Elche, SPAIN

## Abstract

Sucrose-phosphate phosphatase (SPP) catalyses the final step in the sucrose biosynthesis pathway. *Arabidopsis thaliana* genome codifies four SPP isoforms. In this study, the four *Arabidopsis thaliana* genes coding for SPP isoforms have been cloned, expressed in *Escherichia coli* and the kinetic and regulatory properties of the purified enzymes analysed. SPP2 is the isoform showing the highest activity, with SPP3b and SPP3a showing lower activity levels. No activity was detected for SPP1. We propose that this lack of activity is probably due to the absence of an essential amino acid participating in catalysis and/or in the binding of the substrate, sucrose-6-phosphate (Suc6P). The expression patterns of *Arabidopsis SPP* genes indicate that *SPP2* and *SPP3b* are the main isoforms expressed in different tissues and organs, although the non-catalytic SPP1 is the main isoform expressed in roots. Thus, *SPP1* could have acquired new unknown functions. We also show that the three catalytically active SPPs from *Arabidopsis* are dimers. By generating a chimeric SPP composed of the monomeric cyanobacterial SPP fused to the higher plant non-catalytic S6PPc domain (from SPP2), we show that the S6PPc domain is responsible for SPP dimerization. This is the first experimental study on the functionality and gene expression pattern of all the SPPs from a single plant species.

## Introduction

Sucrose is an essential carbohydrate for higher plants and other photosynthetic organisms and considered to be one of the main products of photosynthesis [1, 2]. Sucrose is primarily synthesized in photosynthetic cells and transported to the rest of the plant to provide carbon and energy for growth and for the accumulation of carbon reserves. Besides, sucrose is involved in the regulation of different processes including transcriptional and post-transcriptional control and stress responses [2–8].

Sucrose is synthesised by the consecutive action of sucrose-phosphate synthase (SPS; EC 2.4.1.14) and sucrose-phosphate phosphatase (SPP; EC 3.1.3.24). SPS catalyses the synthesis of Suc-6-P from UDP-glucose and fructose-6-phosphate (Fru6P). In the second step of the pathway, SPP catalyses the irreversible hydrolysis of Suc-6-P to sucrose and displaces the reaction catalysed by SPS in the direction of sucrose synthesis [9, 10]. SPP encoding genes have been described in different plant species such as *Arabidopsis*, tomato, rice, wheat, maize and coffee [11, 12] where they constitute gene families with different number of members depending on the species. However, studies on the biochemical properties of SPP isoforms are scarce and no comprehensive study of all the isoforms from a single species have been done to date. In *Arabidopsis*, four genes show homology to *SPP*, while in wheat and rice three and four genes have been described, respectively [11]. The four genes that code for SPP in *Arabidopsis* display a similar exon-intron structure [2, 11]. *Arabidopsis SPPs* are referred as *SPP1* (At1g51420), *SPP2* (At2g35840), *SPP3a* (At3g54270) and *SPP3b* (At3g52340) [11].

SPP sequences share homology with members of the L-2-haloacid dehalogenase (HAD, http://pfam.sanger.ac.uk/family/PF00702) superfamily of proteins [2, 13–15]. *Arabidopsis* SPPs belong to the subfamily IIB that includes sucrose phosphate phosphatases from plants and cyanobacteria (IPR012847, http://www.ebi.ac.uk/interpro/entry/IPR012847) [16]. The HAD superfamily is characterized by three conserved motifs (I, II and III) related to the active site [13, 17, 18]. The crystal structure of SPP from the cyanobacterium *Synechocystis* sp. PCC 6803 has been elucidated and a catalytic mechanism proposed in which the three typical domains of the HAD proteins are involved in catalysis [13]. SPPs from different plants have been characterized. The enzyme has been shown to be a dimer with a molecular mass of around 100 kDa, formed by subunits of approximately 50 kDa [19–21]. A carboxy-terminal domain of about 160 amino acids is present in higher plant SPPs that has been proposed to participate in dimerization (S6PPc domain), while prokaryotic forms of SPP are monomeric and lack this domain [2]. However, no functional studies have been performed to demonstrate the role of the S6PPc domain in dimerization.

As above referred, the sucrose synthesis pathway involves two enzymatic steps. SPS has been reported as the main regulatory point [3, 22–25], with some isoforms showing overlapping functions [26], while the role of SPP in the control of sucrose synthesis still remains controversial. SPP has not been considered to be rate limiting for sucrose synthesis and, in the case of tobacco, the control coefficient on sucrose synthesis was estimated to be close to zero [23]. In fact, tobacco plants transformed with *SPP* RNAi, with reductions of up to 80% in SPP activity, show almost none or little effect on sucrose synthesis, suggesting that there is no requirement for a 1:1 molar ratio between SPS and SPP [23]. Similar conclusions were obtained in cold-stored potato tubers transformed with *SPP* RNAi [27]. On the other hand, some evidences on enzyme activity indicate that SPP may contribute to sucrose synthesis control [19, 28, 29], role that could be related to the fact that SPP may establish a complex with SPS [30, 31]. In fact, it has recently been shown that SPS and SPP interact *in planta* [32], and this interaction may provide a new level of regulation. Additionally to their role in sucrose synthesis, it has been reported that SPP may have other functions. In this respect, *Arabidopsis* plants overexpressing sorghum SPP show an alteration in seed germination, suggesting a role of SPP in the process [33].

Different studies on the regulation of SPP suggest that sucrose may act as a regulator of the enzyme activity, while in other cases the results are not so evident. It has been reported that SPP activity from partially purified sugar cane and from carrot roots crude extracts is inhibited by sucrose at physiological concentrations (*K*_*i*_ 10 mM) [34]. 100 mM sucrose only partially inhibited the activity in crude extracts from a number of species [35]; i.e. only a 9% inhibition was observed in purified SPP from pea shoots [20]. Also, partially purified SPP from rice and lettuce, showed a weak inhibition of the enzyme activity similar to that observed in pea shoots [21, 36]. In accordance, Lunn *et al*. [19], studying homogeneity-purified SPP from rice, observed that sucrose is a weak competitive inhibitor of SPP with a *K*_*i*_ around 200–400 mM, but the inhibition was slightly potentiated by decreasing concentrations of Suc6P, the substrate of the enzyme.

In this study, the four different *Arabidopsis* SPP cDNAs were cloned and expressed in *Escherichia coli*. The activity and properties of the recombinant enzymes were studied and analysed. A highly conserved Ser was identified as an amino acid residue necessary for catalysis. We also present novel data about the expression pattern of the four *Arabidopsis SPP* genes. Finally, we show that the non-catalytic S6PPc domain is involved in SPP dimerization.

## Materials and Methods

### Plant material and growth conditions

*Arabidopsis thaliana*, Columbia ecotype-0 (Col-0) plants were grown in controlled cabinets on soil under 16 h light / 8 h dark cycle, with temperatures ranging from 22°C (day) to 18°C (night). Seeds were incubated 4 days at 4°C in the dark before sowing. Q-PCR assays were performed using plant material harvested 19 (leaves) or 28 (shoots, flowers, siliques and roots) days after sowing, at Zeitgeber time (ZT) 16.

### Cloning of cDNA and plasmids construction

cDNAs encoding *SPP2* and *SPP3b* were obtained from the RIKEN Genomic Sciences Center, Japan [37, 38]. *SPP1* and *SPP3a* were PCR-amplified from total cDNA preparations from roots or rosette leaves, respectively. cDNA fragments were amplified by PCR using specific primers (Table 1) which were designed with the appropriate restriction sites and a start codon (Met). Next, they were cloned into the pGEM-T Easy vector and finally, in the desired expression vector, which incorporated either a His_6_ (pQE-80L) or a His_10_ tag in the N-terminus (pET-19b). The resulting constructs were individually introduced into *E*. *coli* BL21 (DE3) cells for induction assays and further heterologous recombinant protein expression analysis.

**Table 1:** Primers used in this study.

Primer	Sequence^a^^,^^b^	Construct
P1 (*Bam*HI)	AAATGG**GGATCC**GAGCGGTTAACATCTCCTCCTCG	His_6_-SPP1 (pQE-80L)
P2 (*Pst*I)	AATCGG**CTGCAG**TCAGATGATCCAGTTGCTATCATCC	
P3 (*Nde*I)	GGAATTC**CATATG**GAGCGGTTAACATCTCC	His_10_-SPP1 (pET-19b)
P4(*Bam*HI)	GGG**GGATCC**TCAGATGATCCAGTTGCTATC	
P5 (*Nde*I)	GGAATTC**CATATG**GAGCGTCTAACATCTCC	His_10_-SPP2 (pET-19b)
P6 (*Bam*HI)	GGG**GGATCC**TCAGATGATCCAGCTGCTATC	
P7 (*Nde*I)	GGAATTC**CATATG**GATAGGCTTGAAGGACC	His_10_-SPP3a (pET-19b)
P8 (*Bam*HI)	GGG**GGATCC**TTAGAAAATCCATTTTTCTTG	
P9 (*Nde*I)	GGAATTC**CATATG**GAGCGGCTGATTTCTCA	His_10_-SPP3b (pET-19b)
P10 (*Bam*HI)	GGG**GGATCC**TCAGAGAATCCAAGAACTGTT	
P11 (*Bam*HI)	GGG**GGATCC**TTAAAGCTTGAAGTGACCAAT	His_10_-S6PP (pET-19b)
P12 (*Nde*I)	GGAATTC**CATATG**CCGAACCTTTCTCCAAG	His_10_-S6PPc (pET-19b)
P13 (*Bam*HI)	GGAATTCA**GGATCC**CGACAGTTATTGCTAATTTCTG	His_6_-SynSPP (pQE-80L)
P14 (*Pst*I)	AAACAT**CTGCAG**TTAAGCTTGGCTGCAGGTCG	
P15 (*Spe*I)	GGAATTC**ACTAGT**CTTGGTCCGAACCTTTCTCCAAGA	S6PPc-Ct-fusion
P16 (*Spe*I)	GGAATTC**ACTAGT**GCTCAAAAAATCGAAATGGGCGAT	SynSPP-Nt-fusion
P17	GTGTTTTCTACGGGAAGAGCACCGACATTGTATAAAG	SPP2_S54A_
P18	AAGAAGAGAGTCATGGCGATAAGCGTGTTC	
P19	GTTTTCTCAACAGGAAGATCTCAAACAATGTACAAGA	SPP1_A55S_
P20	AAGAAGAGAGTCGTGTCGATAAGCGTCTTC	

^a^ Restriction sites are highlighted in bold.

^b^ Single base substitutions are marked within squares.

### RNA isolation and Real-Time Q-PCR

RNA was isolated employing Trizol reagent (Invitrogen) and 1 μg used to synthesize cDNA employing the Quantitec Reverse Kit (Qiagen) as described by Ortiz-Marchena et al. [39]. Real-time quantitative PCR (Q-PCR) assays were achieved using Exiqon Universal Library probes as described by Ventriglia et al. [40]. The specific oligonucleotides and Exiqon probes used were: SA648 (5’-tgttgcacaacaactgtcaaat-3’), SA649 (5’-gcacatgttcccacacaaac-3’) and probe #12 for *SPP1*; SA584 (5’-agaagctagcaacttccctgag-3’), SA585 (5’-gctaaccttgtgtggcctct-3’) and probe #124 for *SPP2*; SA661 (5’-ggttcttccagggatattagagg-3’), SA662 (5’-caagtagatatgtcaaagcaccttgt-3’) and #17 for *SPP3a*; SA646 (5’-gaggcattgaccaaggaact-3’), SA647 (5’-ccccaactgtaaattatcttgacat-3’) and probe #67 for *SPP3b*; and SA532 (5’-gaagttcaatgtttcgtttcatgt-3’), SA533 (5’-ggattatacaaggccccaaaa-3’) and probe #119 for ubiquitin. For Real-Time Q-PCR assays, three technical repetitions of samples obtained from three independent experiments were performed.

### Purification of recombinant enzymes

10 ml of an overnight culture of *E*. *coli* BL21 overexpressing the desired SPP gene was added to 1 L of LB medium supplemented with 100 μg/mL ampicillin and subsequently grown at 30°C until the A_600_ reached 0.8. Protein induction was started by addition of 1 mM IPTG and further incubation during 4 h at 30°C. At this stage, cells were harvested by centrifugation and the cell precipitate resuspended in lysis buffer (25 mM HEPES-KOH (pH 7.5), 150 mM KCl, 1 mM PMSF, 8 mM MgCl_2_) and disrupted by sonication. The crude lysate was centrifuged at 60,000 *g* for 30 min at 4°C and the resulting supernatant was used in an affinity purification step employing a HisTrap HP Column (GE Healthcare). Bound proteins were eluted under native conditions by applying a linear gradient from 0% to 100% (v/v) elution buffer (25 mM HEPES-KOH (pH 7.0), 150 mM KCl, 8 mM MgCl_2_, 500 mM imidazole).

Protein was quantified by the Bradford dye-binding method [41] with ovalbumin as standard.

### Molecular mass determination

For further purification and molecular mass determination, chromatographic fractions showing SPP activity were combined and dialyzed against lysis buffer, without Mg^2+^, in the presence of 1 mM PMSF. 0.5 ml fractions were loaded onto a Superose 12 10/300 GL column (GE Healthcare) for size-exclusion chromatography. Elution was performed with the same buffer at a rate of 0.4 ml/min. 0.4 ml fractions were collected and assayed for SPP activity. The molecular mass estimation of the eluted fractions was calculated based on a protein standards calibration curve: β-amylase (β-Amy, 200 kDa), alcohol dehydrogenase (ADH, 150 kDa), bovine serum albumin (BSA, 66 kDa), carbonic anhydrase (CA, 29 kDa) and cytochrome c (Cyt.c, 12.4 kDa).

### Gel electrophoresis and Western blotting

Proteins were separated by SDS-PAGE on 10% or 12% (w/v) polyacrylamide gels as described by Laemmli [42] and stained with Coomassie Blue R-250 or transferred to nitrocellulose membranes. The membranes were then probed with anti-His-tag antibody (Qiagen, Cat No. 34660). Membranes were incubated with the antibody at an 1:1000 dilution in TBS containing non-fat milk 5% (w/v) as blocking agent, washed with TBS buffer plus 0.1% (v/v) Tween 20 and developed with a luminescent assay (WesternBright^™^ Quantum, Advansta).

### Enzyme assays

SPP activity was determined by following the release of Pi from Suc6P. Unless otherwise indicated, the assay mixture contained 25 mM HEPES-KOH (pH 7.0), 8 mM MgCl_2_ and 1.25 mM Suc6P in 1 ml at 37°C. Modifications of the reaction components were made as required in individual experiments. The reaction was initiated by the addition of the enzyme sample and stopped with 54 μl of trichloroacetic acid 6.1 M. Pi released was measured using SnCl_2_-ammonium molybdate reagent [43]. Kinetic parameters (*K*_*m*_ and *kc*_*at*_) were determined from initial velocity data using the nonlinear regression software Anemona.xlt [44]. One unit (U) corresponds to 1 μmol of Pi released per minute.

### Effect of divalent ions and determination of optimum pH

For the study of the effect of divalent ions, purified fractions were dialyzed against 25 mM HEPES-KOH (pH 7.5). Later, the divalent ion of interest was added to a final concentration of 8 mM. The pH dependence of SPP activity was determined at optimum values of divalent ion, temperature and Suc6P concentration. pH-dependent curves were performed using the following buffers: MES (pH 5.5–7.0), HEPES (pH 7.0–8.0), Tris (pH 8.0–9.0), CHES (pH 9.0–10.0) and CAPS (10.0–11.0) at 50 mM final concentration, adjusted to the indicated pH with NaOH or HCl.

### Transitory expression in *Nicotiana benthamiana*

To verify SPP dimerization *in vivo*, *Arabidopsis* SPP2 and the chimeric *Synechocystis SPP* fused to the *S6PPc* domain of *SPP2* were cloned in pYFN43 and pYFC43 to produce fusions to the YTP N-terminal part (YFN-SPP2 and YFN-SynSPP-S6PPc) as well as to the YFP C-terminal part (YFC-SPP2 and YFC-SynSPP-S6PPc) to perform BiFC assays. Specific primers were used for each gene (*SPP2*: 5'-GGGGACAAGTTTGTACAAAAAAGCAGGCTTCATGGAGCGTCTAACATCTCCTCCT-3', 3'-GGGGACCACTTTGTACAAGAAAGCTGGGTCTCAGATGATCCAGCTGCTATCATCC-5'; SynSPP-S6PPc: 5'-GGGGACAAGTTTGTACAAAAAAGCAGGCTTCATGAGAGGATCGCATCACCATCAC-3', 3'-GGGGACCACTTTGTACAAGAAAGCTGGGTCTCAAAGCTTGAAGTGACCAATGGCT-5'). These constructs were introduced into *Agrobacterium tumefaciens* strain GVG3101 pmp90. 4-week old *Nicotiana* plants were agroinfiltrated with the following combinations: YFN-SPP2 and YFC-SPP2 and YFN- SynSPP-S6PPc and YFC- SynSPP-S6PPc. As negative controls, pairs of YFC-SPP2 and YFC-SynSPP-S6PPc with YFN-AKIN10 were used. As positive controls, amino and carboxy parts of AKIN10 were used, following protocols previously described [45, 46]. Fluorescent interactions were visualized under a confocal microscope Leica TCS SP2/DMRE using an excitation wavelength of 514 nm.

### Genome databases

Completed cyanobacterial genomes in GenBank [47] and CyanoBase [48] were searched for ORFs with homology to plant SPP (*Arabidopsis thaliana*) sequences, using the TBLASTN algorithm. The deduced amino acid sequences with E values lower than 1 x 10^−5^ were used to search GenBank non-redundant database using the BLASTP algorithm.

### Reagents and services

Biochemical reagents were purchased from Sigma-Aldrich. Restriction endonucleases were obtained from Takara^™^. Primers were synthetized by IDT. The pGEM-T Easy vector was purchased from Promega. pQE-80L and pET-19b vectors were purchased from Qiagen and Novagen, respectively. Anti-His-tag antibodies were obtained from Qiagen (Cat No. 34660). Sequencing was carried out at The Institute of Parasitology and Biomedicine "López-Neyra" (IPBLN), Spanish National Research Council (CSIC).

## Results and Discussion

### Properties of *Arabidopsis* Sucrose phosphate phosphatases

**Fig 1:**
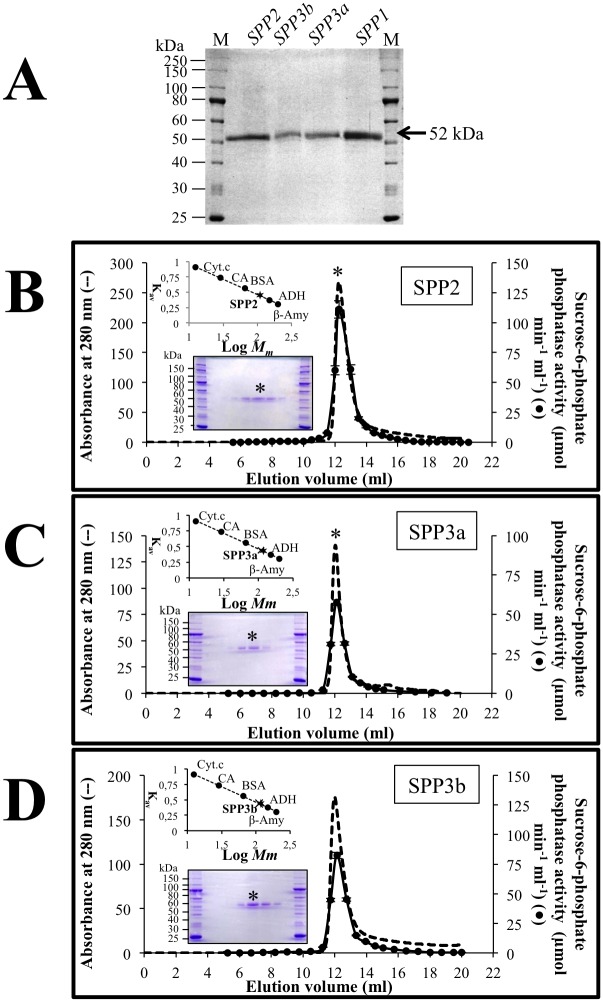
Purification and molecular mass determination of *Arabidopsis* SPP isoforms. (A) SDS-PAGE analysis of purified SPP fractions after gel filtration (Superose 12 10/300 GL). In each case 4 μg of protein were loaded per lane. Purification folds were 306, 109 and 226 for SSP2, SPP3a and SPP3b, respectively. This parameter could not be estimated for SPP1 due to its lack of activity. Proteins were visualized by staining with Coomassie Blue R-250. Lane M, Molecular mass (kDa) markers. Elution profiles of recombinant N_t_-Histag-SPP isoform SPP2 (B), SPP3a (C) and SPP3b (D), applied to a Superose 12 10/300 GL column. A calibration curve is displayed on the upper insert. Molecular mass standards: β-Amy, β-Amylase (200 kDa); ADH, alcohol dehydrogenase (150 kDa); BSA, bovine serum albumin (66 kDa); CA, carbonic anhydrase (29 kDa); and Cyt.c, cytochrome c (12.4 kDa). SDS-PAGE analysis of the fractions around the activity peaks (highest activity fraction marked with an asterisk) is displayed in the lower insert. As observed, both peaks, corresponding to absorbance at 280 nm (broken line) and sucrose-phosphate phosphatase activity (solid line), co-eluted.

Heterologous production of recombinant Nt-Histag *Arabidopsis* SPP1, SPP2, SPP3a and SPP3b were carried out in *E*. *coli* as described in the Materials and Methods section. The recombinant SPPs fused to a His-Tag were purified to a high level (Fig 1*A*). Table 2 summarizes the purification steps followed for SPP2. SPP2 was purified about 306-fold, while SPP3a and SPP3b were purified about 109 and 226-fold (data not shown), respectively. SPPs from *Arabidopsis* showed a molecular mass of about 52 kDa under denaturing conditions (Fig 1*A*). However, when SPP2, SPP3a and SPP3b were analysed by gel filtration under non-denaturing conditions they displayed a molecular mass of about 90 kDa (Fig 1*B*,1*C* and 1*D*), thus indicating that *Arabidopsis* SPPs are dimeric proteins. This is in accordance with previous studies from other higher plants as pea and rice [19–21], but differs from cyanobacterial SPPs that have been shown to be monomeric enzymes [49].

**Table 2:** Purification of Nt-Histag SPP2 expressed in *E*. *coli* (BL21).

Purification steps	Total protein (*mg*)	Yield (*%*)	Total activity^a^ (*U*)	Specific activity (*U/mg protein*)	Purification (*fold*)
Crude supernatant	1252	100	164.01	0.131	1
Ni-NTA column	4.8	0.3	107.40	22.376	171
Concentration^b^	3.6	0.2	78.19	21.721	166
Superose 12	1.2	0.1	48.04	40.039	306

^a^ One unit (U) is defined as the hydrolysis of 1 μmol of sucrose-6-phosphate/min at 30°C.

^b^ Ni-NTA fractions were pooled and concentrated to 0.25 ml with an Amicon Ultra-3K concentrator.

Even in the pure fractions, we could not detect any activity for the recombinant SPP1 isoform. Among the active *Arabidopsis* SPPs, SPP2 was shown to be the enzyme with the highest activity, while SPP3a and SPP3b showed lower activity levels (Table 3): SPP3b activity was about 20-times lower than SPP2, while SPP3a displayed about 200-times less activity. When we tested the affinity for Suc6P of the three active SPPs (Table 3), SPP2 was shown to have the lowest *Km* for Suc6P (0.73 mM), SPP3a showed a similar *Km* value (0.87 mM), while SPP3b displayed a 5-times higher *Km* (3.46 mM) than SPP2. Taken together, these results suggest that SPP2 is the main isoform responsible for sucrose synthesis in *Arabidopsis* with the highest activity and affinity for the substrate, while SPP3a is the isoform with the lowest activity and catalytic efficiency for the substrate (Table 3).

**Table 3:** Kinetic properties of *Arabidopsis* SPP isoforms.

	SPP2	SPP3a	SPP3b
*K*_m_ S6P (mM)	0.73 ± 0.32	0.87 ± 0.39	3.46 ± 0.71
SA_max_ (μmol min^-1^ mg^-1^ prot)^a^	40.04 ± 7.31	0.22 ± 0.04	2.09 ± 0.28
*k*_*cat*_ (min^-1^)	4066.36	23.75	215.32
*k*_*cat*_ /*K*_m_ (min^-1^ mM^-1^)	5551.34	27.26	62.18

^a^ The limit of detection was approximately 0.4 nmol min^-1^ mg^-1^ prot.

**Fig 2:**
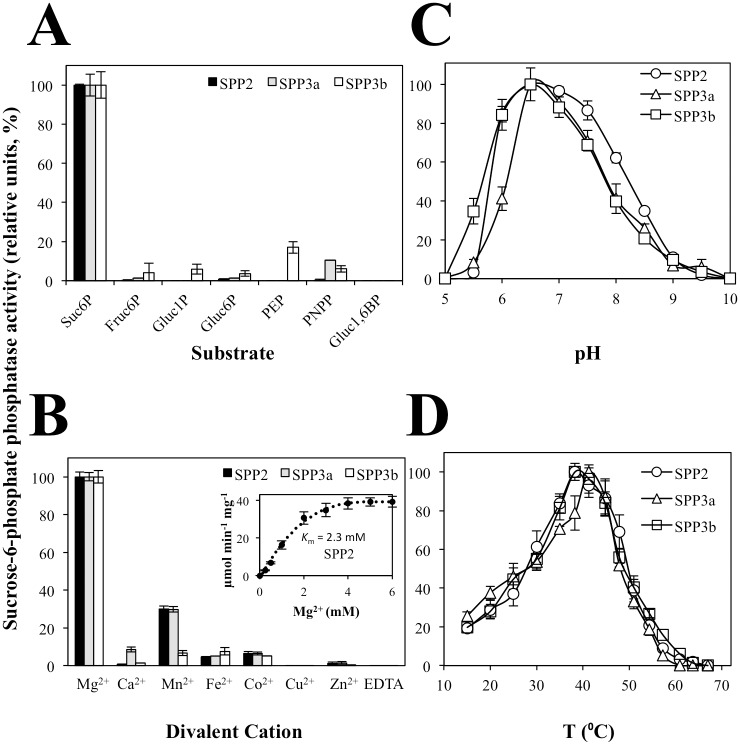
Biochemical characterization of recombinant *Arabidopsis* SPP isoforms. Substrate dependence, metal cofactor specificity, as well as the optimum temperature and pH of *Arabidopsis* SPP isoforms are shown. (A) SPP activity using different sugar-phosphates as substrate. 100% activity corresponds to 37.68±0.18, 0.14±0.01 and 1.93±0.05 μmol min^-1^ mg^-1^ prot for SPP2 (black column), SPP3a (grey column) and SPP3b (white column) with Suc6P as substrate, respectively. (B) SPP activity was determined in the presence of 8 mM divalent cation. 100% activity is determined in the presence of optimum Mg^2+^ concentration (5 mM) and corresponds to 38.13±0.92, 0.14±0.00 and 1.92±0.04 μmol min^-1^ mg^-1^ prot for SPP2, SPP3a and SPP3b as in (A), respectively. Insert shows the Mg^2+^ dependence of SPP2 activity. (C) SPP activities were estimated at different pH using a combination of buffers as described in the Materials and Methods section. 100% activity corresponds to 36.35±0.54, 0.13±0.01 and 1.90±0.01 μmol min^-1^ mg^-1^ prot for SPP2 (open circle), SPP3a (open triangle) and SPP3b (open square), respectively. (D) Effect of temperature on the activity of *Arabidopsis* SPP isoforms. 100% specific activity corresponds to 37.21±0.61, 0.14±0.01 and 1.92±0.06 μmol min^-1^ mg^-1^ prot for SPP2, SPP3a and SPP3b as in (C) respectively. Data were obtained from three independent experiments and are shown as means ± S.D. Fruc6P, fructose-6-phosphate; Gluc1P, glucose-1-phosphate; Gluc6P, glucose-6-phosphate; Gluc1,6BP, glucose-1,6-bisphosphate; Suc6P, sucrose-6-phosphate; PEP, phosphoenol pyruvate; PNPP, p-nitrophenyl phosphate.

Studies of SPP from different organisms show that the enzyme has a relatively high specificity for Suc6P [49]. Therefore, we tested the specificity of the active SPPs from *Arabidopsis* for different substrates. In agreement with previous studies in other species [49], Fruc6P, Gluc1P, Gluc6P, PEP, p-nitrophenyl phosphate (PNPP) and Gluc1,6BP were poor substrates for SPPs (Fig 2*A*) and only in the case of SPP3b, PEP could account for about 15% of the activity obtained for Suc6P as substrate. It seems thus clear that SPPs are enzymes that show a high affinity for suc6P and very low for other phosphorylated sugars.

It has been previously shown that SPP activity is strictly dependent on the presence of Mg^2+^. Accordingly, the purification of the *Arabidopsis* SPPs including a dialysis step in a buffer without divalent cations resulted in enzyme preparations with no activity. Fig 2*B* shows that activity of SPP2, SPP3a and SPP3b is dependent on the presence of a divalent cation in the reaction buffer, the maximal activity being observed in the presence of Mg^2+^, as previously described for rice and pea SPPs [19–21]. In the presence of other divalent cations, *Arabidopsis* SPP activity reached very low levels (about 5% of maximal activity), except for SPP2 and SPP3b that in the presence of Mn^2+^ exhibited 30% of their maximal activity (Fig 2*B*). Fig 2*B* insert shows that SPP2 maximal activity was obtained in the presence of 5 mM Mg^2+^, with a *K*_*m*_ for Mg^2+^ of 2.3 mM, in the range reported for the rice enzyme [21]. The high preference of SPP for Mg2+, which can only be partially replaced by Mn2+, appears to be a common property of the enzyme in all organisms investigated to date [21, 36, 50].

*Arabidopsis thaliana* SPPs showed their maximal activity at neutral pH (Fig 2*C*). As reported for rice, lettuce and sugar cane [21, 36, 51], the activity of the three active *Arabidopsis* SPPs decreased sharply at more acidic pHs, with no activity remaining at pH 5, while at more basic pH the activity was detectable up to pH 9–9.5 (Fig 2*C*). On the other hand, maximal activity of SPPs was observed at temperatures ranging between 35–45°C (Fig 2*D*).

**Fig 3:**
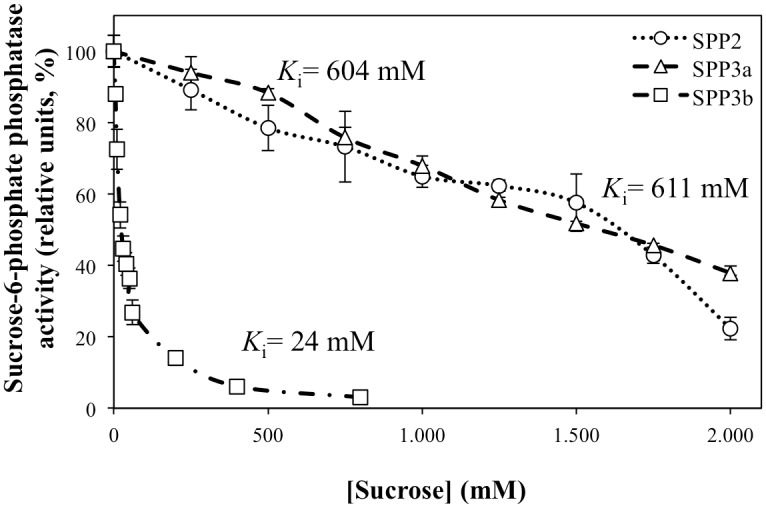
Effect of sucrose on *Arabidopsis* SPP activity. SPP activities were determined with 1.25 mM Suc6P using enzyme samples preincubated for 15 min at 37°C in the presence of increasing concentrations of sucrose. 100% activity corresponds to 37.43±0.65, 0.13±0.04 and 0.89±0.01 μmol min^-1^ mg^-1^ prot for SPP2 (open circle), SPP3a (open triangle) and SPP3b (open square), respectively. Data were obtained from three independent experiments and are shown as means ± S.D. The calculated *K*_i_ is indicated above each activity curve.

In some species, it has been described that SPP is inhibited by sucrose, while in others it has no effect or acts as a weak competitive inhibitor [13, 19–21, 34–36, 50]. We have analysed the effect of sucrose on purified *Arabidopsis* SPPs and observed that this sugar differentially inhibited *Arabidopsis* SPP isoforms. Sucrose was a weak inhibitor of SPP2 and SPP3a (Fig 3), while SPP3b was the most sensitive isoform (*K*_*i*_ of 24 mM). SPP2 and SPP3a inhibition by sucrose required much higher concentrations (*K*_*i*_ 611 mM for SPP2, *K*_*i*_ 604 mM for SPP3a) than for SPP3b, suggesting that under physiological conditions SPP2 and SPP3a are not inhibited by sucrose. In this sense, sucrose could bind to the active site of the SPP enzyme in a position similar to the substrate Suc6P as a competitive inhibitor [13]. In our hands, SPP2 and SPP3a activities were slightly inhibited by sucrose concentrations around 100 mM in the presence of 1.25 mM Suc6P in the reaction assay mixture (Fig 3), with *K*_i_ around 600 mM. These results are comparable to those reported for pea shoots [20], rice leaves [21] and lettuce leaves [36]; but differ from those for sugar cane SPP [34], that was significantly inhibited (60%) at 50 mM sucrose concentration. Similarly to sugar cane SPP, SPP3b showed a Ki for sucrose of 24 mM. Thus, we have observed two types of responses to sucrose inhibition for *Arabidopsis* SPPs isoforms. Concentrations required for SPP2 and SPP3a inhibition by sucrose are much higher than those physiologically occurring [52], except perhaps in sink organs such as sugar cane shoots or carrot roots, while SPP3b might be regulated by sucrose in physiological conditions.

### Recovery of *Arabidopsis* SPP1 activity

**Fig 4:**
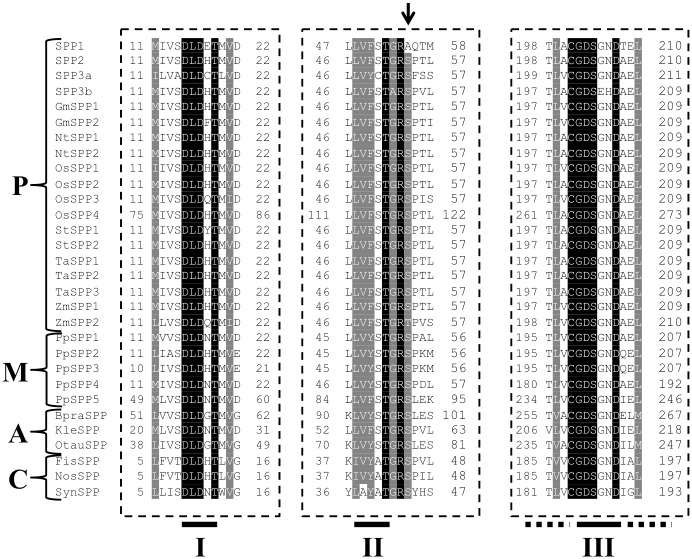
Comparison of amino acid sequences of *Arabidopsis* SPP isoforms and SPPs from diverse organisms. Multiple-sequence alignment of the deduced amino acid sequences of the *Arabidopsis* SPP isoforms with SPPs from different species is shown. Sequences were aligned with the CLUSTAL Omega program [61] using a BLOSUM matrix. Identical residues and residues widely conserved are highlighted in black and light grey, respectively. Conserved residues in motifs I, II, and III of the HAD-type phosphatases are shown underlined. An arrow points to the non-conservative substitution of the widely conserved Ser in motif II by Alanine in the sequence of the *Arabidopsis* SPP1. P, higher plants; M, mosses; A, green algae; C, cyanobacteria. Accession numbers are indicated in S1 Table, except for OsSPP1 (Q94E75); OsSPP2 (Q6YXW6); OsSPP3 (B9FME4); OsSPP4, (B9F2N9) and KleSPP, (G1UJV3).

As indicated in the Introduction section, SPP protein sequences share homology with members of the L-2-haloacid dehalogenase (HAD, IPR006379) superfamily of proteins [13], with the *Arabidopsis* SPPs included in the family of Sucrose Phosphate Phosphatases from plants and cyanobacteria (IPR012847). The HAD superfamily is characterized by three conserved motifs (I, II and III) related to the active site [13, 17, 18]. To determine the basis for the lack of activity of SPP1, a sequence comparison of the three motifs from different organisms was performed (Fig 4). All three motifs are highly conserved among plant, algae, cyanobacteria and mosses. To date, the only structural study of an SPP has been carried out in the cyanobacterium *Synechocystis* sp. PCC 6803 [13, 14]. The *Synechocystis* SPP structure is composed of two domains, a core domain containing the catalytic site, and a smaller cap domain that contains a glucose-binding site. The three conserved HAD motifs in SPP form the active site and are located at the interface between both domains, which are connected by two hinge loops that allow the binding of Suc6P [14]. The phosphate group of Suc6P interacts with Lys-163, Asp-9, Gly-42 and Thr-41 [13]. Two of them, Thr-41 and Gly-42, are close to Ser-44 in *Synechocystis* SPP and are highly conserved in plant SPPs (Fig 4). Serine residue 44 in *Synechocystis* is sterically close to threonine 41 (Thr-41), which has been implicated in the establishment of a hydrogen bond with Suc6P and a water molecule during catalysis [13]. *Synechocystis* Ser-44 corresponds to Ser-54 in *Arabidopsis* SPP2, SPP3a and SPP3b, while *Arabidopsis* SPP1 presents an Alanine (Ala-55) at the corresponding position instead of a Serine (Indicated by an arrow in Fig 4). Therefore, this amino acid change could be affecting the interaction with the substrate in the catalytic site and cause SPP1 lack of activity. To check this hypothesis SPP2 Ser-54 residue was changed by PCR to Ala to generate SPP2_S54A_. The mutated cDNA was expressed in *E*. *coli* and indeed the purified SPP2_S54A_ protein showed no activity, suggesting an implication of Ser-54 in SPPs catalysis (Table 4). Likewise, the non-catalytic SPP isoform SPP1 was turned into a catalytic SPP by the substitution of its original Ala-55 with the correspondent, highly conserved Ser, to generate SPP1_A55S_. As shown in Table 4, SPP1_A55S_ is catalytically active, although the level of activity was low.

**Table 4:** Reconstitution of SPP1 activity by site-directed mutagenesis.

Isoform	Residue mutated	Specific activity^a^ (*μmol min*^*-1*^ *mg*^*-1*^ *prot*)
SPP1	none	Not detected
SPP1	A55S	0.02 ± 0.001
SPP2	none	40.04 ± 7.31
SPP2	S54A	Not detected

^a^ The limit of detection was approximately 0.4 nmol min^-1^ mg^-1^ prot.

**Fig 5:**
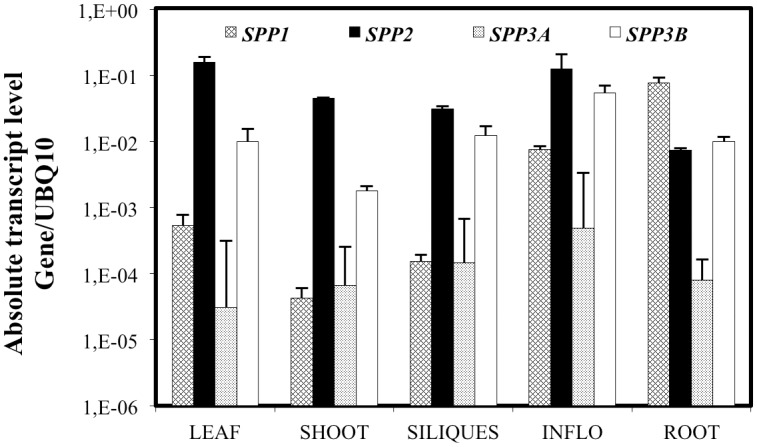
Expression profile of the *Arabidopsis* SPPs encoding genes. mRNA levels of all the *Arabidopsis* SPP-encoding genes were determined by Q-PCR as described under Materials and Methods. In the figure, quantities are represented in a logarithmic plot in order to compare the data among the different genes. The values represent the average of three technical repetitions of samples obtained from three independent experiments. The error bars in the plot represent the S.D.

The presence of an Ala at position 55 in SPP1 could likely affect the optimum orientation of core residues for catalysis and, as a consequence, SPP1 would be unable to hydrolyse Suc6P. However, the fact that the recovery of SPP1 activity is only partial suggests that modifications in other positions may contribute to the lack of activity of this isoform. The presence of members with unknown function (sometimes proposed to be pseudo-genes) in gene families involved in carbon metabolism has been reported before [53, 54]. In some cases additional functions different from their original catalytic activity, for example as transcriptional regulators, have been suggested [55]. In the case of sorghum SPPs, it has been proposed that, at least, one isoform might be involved in seed germination [33]. Thus, we can not exclude that SPP1 could have a different unknown role or that the lack of activity could be due to an strict dependence on its interaction with SPS, as it has been shown that SPPs interact with SPSs [32]. It is worth mentioning that sequence comparison of SPP coding genes from *Brasicaceae* shows that the presence of the non-conserved Ala at position 55 or equivalent is relatively common (data not shown). So, in *Capsella rubella*, *Arabidopsis lyrata* and *Arabidopsis thaliana* one out of four genes show the non-conserved Ala, while in *Brassica napus* and *Brassica rapa*, this amino acid substitution is present in two out of 16 and one out of 8 SPPs, respectively. Considering this, the fact that *SPP1* is the most highly expressed *SPP* isoform in roots and that it is highly expressed in inflorescences (see Fig 5), we cannot rule out the possibility that SPP1, and other SPPs showing an Ala at position 55 or equivalent, could have an alternative function. Further studies are required to reveal the role, if any, of SPP1.

### Expression pattern of *Arabidopsis thaliana SPPs*

We have determined the steady-state mRNA levels of the four *Arabidopsis SPP* genes by Q-PCR. Specific primers for the *SPPs* genes and for the housekeeping gene, *Ubiquitin*-10 [56], were designed (Materials and Methods) and their efficiency and specificity checked. The fragments amplified by the primers were cloned and used as external calibration standards [57]. *SPP* mRNA levels were analysed in leaves, stems, inflorescences, fruits and roots of mature plants (Fig 5). All four *SPP* genes were expressed in the different tissues studied, with *SPP2* showing the highest level of expression among the aerial parts of the plant, followed by *SPP3b*. Expression of *SPP1* and *SPP3a* was about 2 to 3 orders of magnitude lower than *SPP2* levels. *SPP1* showed intermediate levels of expression in aerial tissues, while it was the main isoform expressed in roots.

*SPP2*, which encodes the subunit with the highest catalytic activity, is also the most expressed active isoform in all tissues, suggesting that it accounts for most of the SPP activity in *Arabidopsis* plants, while *SPP3a* expression levels are lower in all tissues assayed (Fig 5). This fact suggests that dephosphorylation of Suc6P by SPP2 and SPP3a is not probably regulated by sucrose in *Arabidopsis* (see Fig 3). On the other hand, *SPP3b* is expressed at relatively high levels in most tissues, so it would be possible that sucrose could regulate SPP3b activity. However, the specific activity determined for SPP3b is about 20 times lower than that determined for SPP2, implying that sucrose concentration may not significantly control the overall SPP activity in the plant.

### Implication of plant S6PPc domain in SPP dimerization

**Fig 6:**
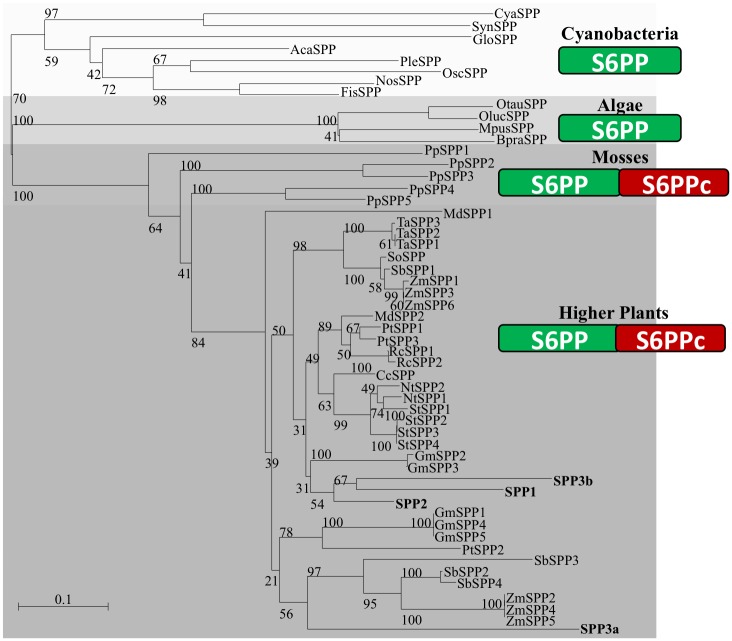
Phylogenetic tree of SPPs in photosynthetic organisms. Unrooted neighbour-joining phylogenetic tree of amino acid sequences of SPPs from diverse organisms is shown. Predicted SPP sequences were obtained from public databases (GenBank, JGI genome databases and InterPro EMB-EBI). Phylogenetic tree was constructed with Seaview software excluding all gaps in the multiple alignment. Values above lines show bootstrap percentages (based on 1,000 replicates). Scale bar indicates number of changes per unit length. SPPs were separated into four main groups: Cyanobacteria, algae, mosses, and higher plants. The domain structure of the putative SPP of each group is displayed as predicted by Pfam database.

**Fig 7:**
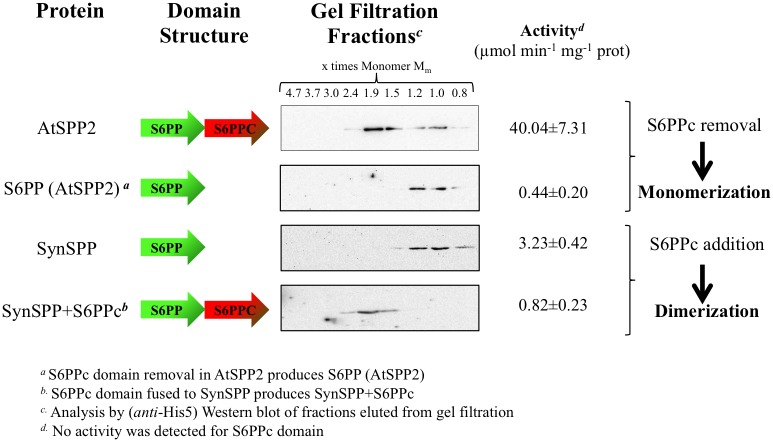
Analysis of the role of S6PPc domain. Schematic representation of the constructs used for the dimerization assays. SPP2, S6PP domain of SPP2, *Synechocystis* SPP and Chimeric *Synechocystis* SPP (SynSPP+S6PPc) were analysed for dimerization and activity. S6PPc domain was fused to SynSPP by the carbonyl group. Proteins were purified as described in Materials and Methods and subsequently applied onto a gel filtration column. Eluted fractions were immunoblotted using anti-His5 antibodies. The activity of each protein or chimera was determined as described in Materials and Methods. Full size Western blots are shown in S1 Fig.

**Fig 8:**
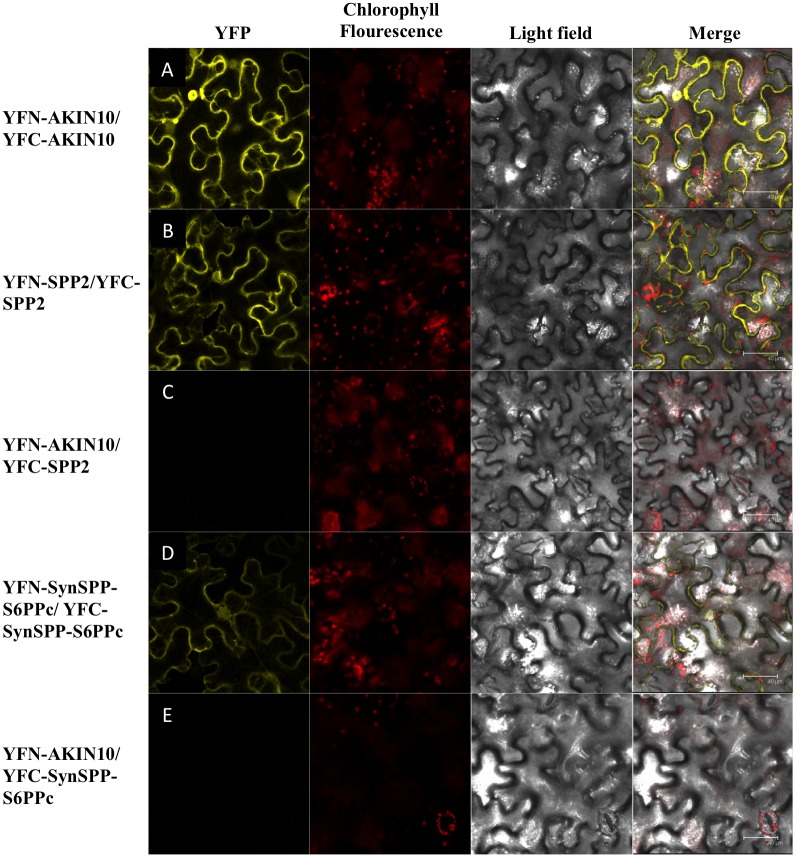
In vivo analysis of SPP dimerization by BiFC. Confocal images of *Nicotiana* leaf cells showing SPP2 and SynSPP-S6PPc dimerization by BiFC assays. (A) YFN-AKIN10/YFC-AKIN10 (positive control); (B) YFN-SPP2/YFC-SPP2; (C) YFN-AKIN10/YFC-SPP2 (negative control); (D) YFN-SynSPP-S6PPc/YFC-SynSPP-S6PPc; and (E) YFN-AKIN10/YFC-SynSPP-S6PPc (negative control). Samples were excited at 514 nm and all images were taken in the same conditions. The white bar in the merge image represents 40 μm.

A search of orthologous *SPP* genes in genomes from several photosynthetic organisms was carried out. In the case of cyanobacteria, the *SPP* gene from *Synechocystis* sp. PCC 6803 was used as query to search the genomes from members of the five cyanobacteria classes defined in Rippka’s classification [58]. According to the results, a total of 49 ORFS (18 Chroococcales, 17 Nostocales, 9 Oscillatoriales, 3 Pleurocapsales, 1 Gloeobacteria, and 1 Stigonematales) showed significant homology to *Synechocystis SPP*. Only three of them have previously been reported as functional SPPs [13, 59, 60], the rest remains as predicted proteins. The genomes of algae, mosses and higher plants were also included in this study. A BLAST comparison identified 173 eukaryotic ORFS with significant hits (5 green algae, 5 bryophyta and 163 tracheophyta). Fig 6 shows a phylogenetic tree of SPPs from cyanobacteria, algae, mosses and higher plants (see S1 Table). SPPs from cyanobacteria and algae have been shown to be monomeric, in contrast with the dimeric nature of higher plant SPPs. As determined from their deduced amino acid sequences, cyanobacterial and algal SPPs lack an extensive C-terminal domain (S6PPc) shared by plant SPPs (Fig 6). Because of its absence in monomeric SPPs, it has been suggested that this extra C-terminal domain is a domain responsible for SPP dimerization. To evaluate this hypothesis, the S6PPc domain from *Arabidopsis* SPP2 (19.9 kDa) was fused to the C-terminus of the monomeric SPP from *Synechocystis* (28.4 kDa) (see Materials and Methods). The chimeric protein (47.9 kDa) was proved to be dimeric by Western blot analysis of gel filtration chromatography fractions (Fig 7) and showed SPP activity. Fig 8 show BiFC assays indicating that SPP2 and the chimeric Synechocystis SPP fused to the S6PPc domain are able to form dimers *in vivo*, thus confirming the role of S6PPc domain in dimerization. In contrast, the removal of the S6PPc domain from *Arabidopsis* SPP2 produced a monomeric enzyme of about 30 kDa (Fig 7) with an activity significantly lower than the native protein.

As indicated before, higher plant SPPs contain a HAD domain and a C-terminal domain, while prokaryotic forms of SPP are monomeric and contain only the HAD domain [11]. The S6PPc domain of the plant enzyme does not show any significant homology with other protein of known function. However, the sequence of a partial cDNA clone from the bryophyte (moss) *Physcomitrella patens* (GenBank accession no. AW497133) would encode a protein showing 57% identity with maize SPP, extending into this C-terminal region. This suggests that acquisition of the C-terminal extension was an early event in the evolution of SPPs in plants. *Synechocystis* SPP shows homology only with the N-terminal region (HAD domain) of the plant enzyme [19] and their kinetic properties are similar: both have similar optimum pH, are specific for Suc6P and are Mg^2+^-dependent [19]. As shown in Figs 7 and 8, the fusion of the SPP2 S6PPc domain to the *Synechocystis* SPP changes the monomeric enzyme into a dimeric active form as observed by gel filtration analysis of the recombinant protein and by BiFC assays. The fact that SPP2 is converted into a monomeric form by eliminating the S6PPc domain, and that the monomeric Synechocystis SPP fused to the S6PPc domain is dimeric, strongly suggest that the S6PP6c domain of higher plants SPPs is responsible for dimerization and might positively affect SPP activity. However, we cannot exclude the possibility that removal of the S6PPc domain could originate a decrease in the activity of SPP2 due to an alteration of the enzyme structure/properties.

## Conclusions

We have characterized the kinetic, regulatory properties and the expression pattern of the SPP family from *Arabidopsis thaliana*. We show that SPP1 is a non-active enzyme, while SPP2 is the most active isoform and show the highest level of expression in aerial parts of the plant. We propose that the lack of SPP1 activity is at least in part related to an amino acid substitution near the active site, although we cannot exclude that SPP1 has another function or need to interact with SPS to be active. Finally, we demonstrate that the S6PPc domain, specific to higher plants SPPs, is responsible for SPP dimerization.

## Supporting Information

S1 Fig Analysis by Western blotting of the fractions eluted from gel filtration of the His-tagged monodomain and bidomain SPP proteins. Fractions were resolved by SDS-PAGE (10 w/v for *Arabidopsis* SPP2 and *Synechocystis* SPP fused to S6PPc domain of *Arabidopsis* SPP2 and 12% w/v for S6PP domain of *Arabidopsis* SPP2 and for *Synechocystis* SPP). Fractions were transferred onto a nitrocellulose membrane and probed with a specific anti-His-tag antibody (Qiagen, Cat No. 34660). Number-average degree of polymerization is given on top of each blot.
(TIFF)

S1 Table Amino acid sequences of the different SPPs displayed in phylogenetic tree of Fig 6. (PDF)
