# Supplementary figures and images for: Characterization of the Sucrose Phosphate Phosphatase (SPP) Isoforms from Arabidopsis thaliana and Role of the S6PPc Domain in Dimerization
Source: PLoS One. 2016 Nov 17;11(11):e0166308. doi: 10.1371/journal.pone.0166308 (PMC5113954; doi:10.1371/journal.pone.0166308)

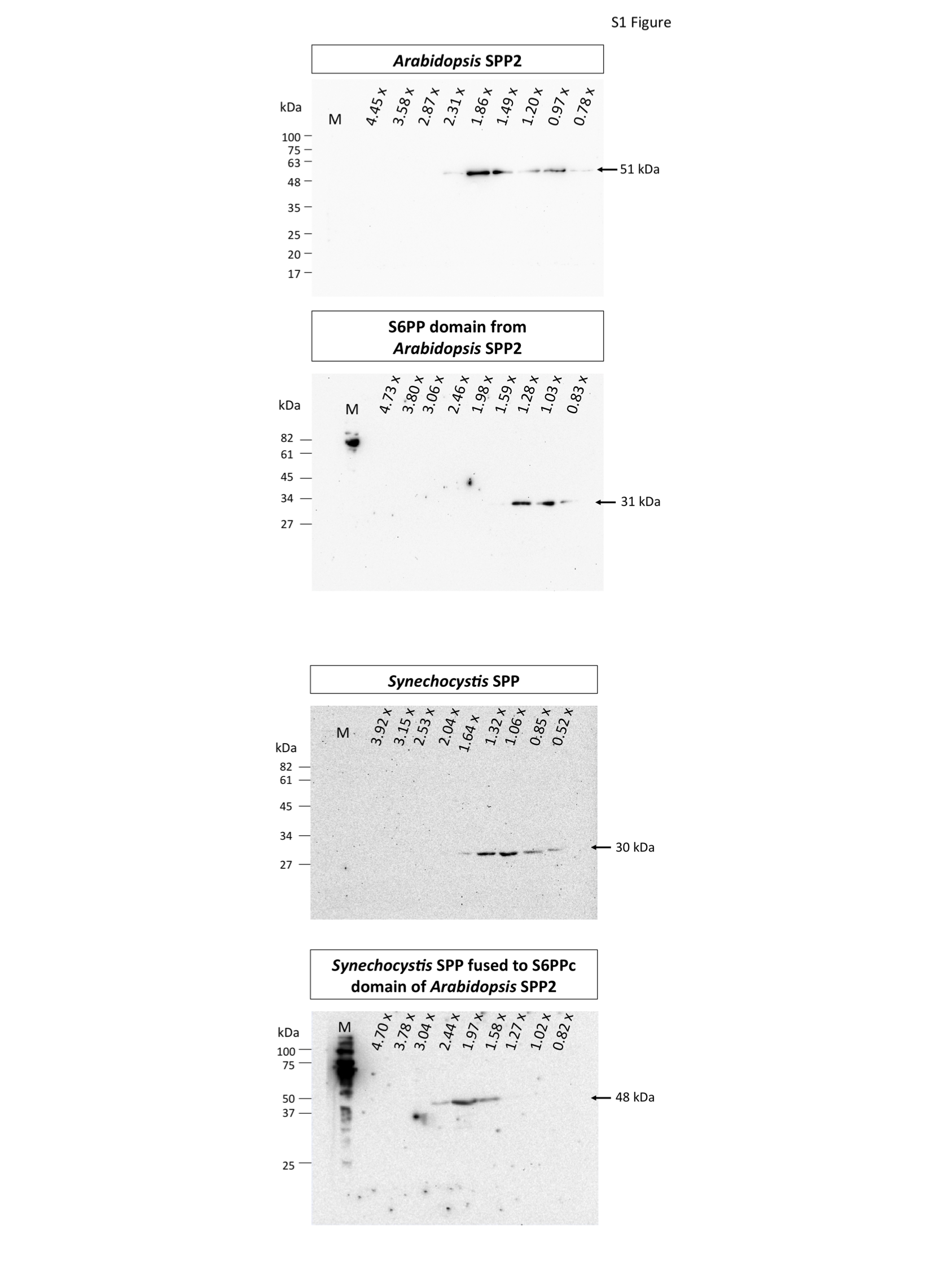

Supplement: S1 Fig — Fractions were resolved by SDS-PAGE (10 w/v for Arabidopsis SPP2 and Synechocystis SPP fused to S6PPc domain of Arabidopsis SPP2 and 12% w/v for S6PP domain of Arabidopsis SPP2 and for Synechocystis SPP). Fractions were transferred onto a nitrocellulose membrane and probed with a specific anti-His-tag antibody (Qiagen, Cat No. 34660). Number-average degree of polymerization is given on top of each blot. (TIFF) [file pone.0166308.s001.tiff]
