# Supplementary material for: Characterization of the Sucrose Phosphate Phosphatase (SPP) Isoforms from Arabidopsis thaliana and Role of the S6PPc Domain in Dimerization
Source: PLoS One. 2016 Nov 17;11(11):e0166308. doi: 10.1371/journal.pone.0166308 (PMC5113954; doi:10.1371/journal.pone.0166308)
Supplement: S1 Table — (PDF) [file pone.0166308.s002.pdf]

S1 Table. Amino acid sequences of the different SPPs displayed in phylogenetic tree of Figure 6.

Protein	UniProtKB[a]	Organism	Phylogeny	Reference
CyaSPP	B8HKQ6	*Cyanothece* sp. PCC 7425	Cyanobacteria	
SynSPP	Q7BII3	*Synechocystis* sp. PCC 6803	Cyanobacteria	(19)
PleSPP	K9T8Y4	*Pleurocapsa* sp. PCC 7327	Cyanobacteria	
OscSPP	K9TSQ1	*Oscillatoria acuminata* PCC 6304	Cyanobacteria	
NosSPP	Q8YZT1	*Nostoc* sp. PCC 7120	Cyanobacteria	
FisSPP	G6FWC7	*Fischerella* sp. JSC-11	Cyanobacteria	
GloSPP	Q7NEP0	*Gloeobacter violaceus PCC 7421*	Cyanobacteria	
AcaSPP	B0CAE8	*Acaryochloris marina MBIC 11017*	Cyanobacteria	
MpusSPP	C1N9B1	*Micromonas pusilla CCMP1545*	Chlorophyta	
OtauSPP	Q01GF9	*Ostreococcus tauri*	Chlorophyta	
OlucSPP	A4RR69	*Ostreococcus lucimarinus*	Chlorophyta	
BpraSPP	K8EHT2	*Bathycoccus prasinos*	Chlorophyta	
PpSPP1	Q45FX0	*Physcomitrella patens* subsp. *patens* (Moss)	Bryophyta	(11)
PpSPP2	A9T1H8	*Physcomitrella patens* subsp. *patens* (Moss)	Bryophyta	(11)
PpSPP3	A9S9K4	*Physcomitrella patens* subsp. *patens* (Moss)	Bryophyta	(11)
PpSPP4	A9TQV3	*Physcomitrella patens* subsp. *patens* (Moss)	Bryophyta	(11)
PpSPP5	A9RNF9	*Physcomitrella patens* subsp. *patens* (Moss)	Bryophyta	(11)
CcSPP	A7IZK6	*Coffea canephora* (Robusta coffee)	Tracheophyta	
GmSPP1	C6T8I9	*Glycine max* (Soybean)	Tracheophyta	
GmSPP2	C6TK02	*Glycine max* (Soybean)	Tracheophyta	
GmSPP3	I1L9P5	*Glycine max* (Soybean)	Tracheophyta	
GmSPP4	I1NED3	*Glycine max* (Soybean)	Tracheophyta	
GmSPP5	I1NED5	*Glycine max* (Soybean)	Tracheophyta	
MdSPP1	Q5J3N9	*Malus domestica* (Apple)	Tracheophyta	
MdSPP2	Q5J3P0	*Malus domestica* (Apple)	Tracheophyta	
NtSPP1	Q5IH14	*Nicotiana tabacum* (Common tobacco)	Tracheophyta	(23)
NtSPP2	Q5IH13	*Nicotiana tabacum* (Common tobacco)	Tracheophyta	(23)
PtSPP1	B9H9N0	*Populus trichocarpa* (Western balsam poplar)	Tracheophyta	
PtSPP2	B9HKI9	*Populus trichocarpa* (Western balsam poplar)	Tracheophyta	
PtSPP3	B9IIH1	*Populus trichocarpa* (Western balsam poplar)	Tracheophyta	
RcSPP1	B9SDM9	*Ricinus communis* (Castor bean)	Tracheophyta	
RcSPP2	Q4FCW2	*Ricinus communis* (Castor bean)	Tracheophyta	
SbSPP1	C5XSH9	*Sorghum bicolor* (Sorghum)	Tracheophyta	
SbSSP2	C5Z001	*Sorghum bicolor* (Sorghum)	Tracheophyta	
SbSPP3	C5Z002	*Sorghum bicolor* (Sorghum)	Tracheophyta	

SbSPP4	V5M3J8	*Sorghum bicolor* (Sorghum)	Tracheophyta	
SoSPP	Q4FCW1	*Saccharum officinarum* (Sugarcane)	Tracheophyta	
StSPP1	A7LH88	*Solanum tuberosum* (Potato)	Tracheophyta	(27)
StSPP2	A7LH87	*Solanum tuberosum* (Potato)	Tracheophyta	(27)
StSPP3	M1CQ12	*Solanum tuberosum* (Potato)	Tracheophyta	
StSPP4	M1CQ13	*Solanum tuberosum* (Potato)	Tracheophyta	
TaSPP1	Q9AXK6	*Triticum aestivum* (Wheat)	Tracheophyta	
TaSPP2	Q9AXK5	*Triticum aestivum* (Wheat)	Tracheophyta	
TaSPP3	Q9ARG8	*Triticum aestivum* (Wheat)	Tracheophyta	
ZmSPP1	Q9FQ11	*Zea mays* (Maize)	Tracheophyta	(19)
ZmSPP2	Q84ZX8	*Zea mays* (Maize)	Tracheophyta	(11)
ZmSPP3	B4FDG9	*Zea mays* (Maize)	Tracheophyta	
ZmSPP4	C0P5Y2	*Zea mays* (Maize)	Tracheophyta	
ZmSPP5	K7TRN1	*Zea mays* (Maize)	Tracheophyta	
ZmSPP6	K7VW75	*Zea mays* (Maize)	Tracheophyta	
SPP1	Q9C8J4	*Arabidopsis thaliana* (Mouse-ear cress)	Tracheophyta	This study
SPP2	Q9SJ66	*Arabidopsis thaliana* (Mouse-ear cress)	Tracheophyta	This study
SPP3a	Q93WU4	*Arabidopsis thaliana* (Mouse-ear cress)	Tracheophyta	This study
SPP3b	Q93XN8	*Arabidopsis thaliana* (Mouse-ear cress)	Tracheophyta	This study

[a] Most of the SPP proteins listed are putative and were selected based on the S6PP-S6PPc domains assignation recorded in the UniProtKG database.
